# Co‐Designing a Multimodal Wearable System for Dementia Care: A Stakeholder‐Informed Mixed‐Methods Study Protocol

**DOI:** 10.1002/alz70858_104232

**Published:** 2025-12-25

**Authors:** Pallavi Nair, Marek Żyliński, Cornelia Junghans, Benedict Hayhoe

**Affiliations:** ^1^ Imperial College London, London, London, United Kingdom

## Abstract

**Background:**

Technological advancements in wearable devices are unlocking new opportunities for real‐time, continuous monitoring of physiological parameters in everyday settings. Among these, in‐ear devices, or hearables, offer unique advantages, including non‐invasiveness, unobtrusiveness, and reduced motion artifacts allows for monitoring parameters such as brain activity, heart rate and movement. While hearables have been explored in other health contexts, their potential for dementia care remains underexplored.

**Method:**

This study protocol outlines a stakeholder‐informed mixed‐methods approach to co‐design a multimodal wearable system tailored for dementia care. The project aims to incorporate the perspectives of key stakeholders— individuals with dementia, their carers and healthcare professionals; General Practitioners (GPs), Adult Social Care staff —to ensure that the technology is accessible, acceptable, and clinically relevant. By enabling the continuous collection of physiological data, this system will complement traditional cognitive assessments and enhance personalized care in both community and clinical settings. The study comprises four interconnected workstreams (WS): Information gathering: WS1‐ review summarising evidence of key physiological parameters for dementia and ethics approval. WS2‐ stakeholder codesign workshops will explore perspectives on the usability, feasibility, acceptability and selection of commercially available hearables. Demo Development and Testing: WS3‐ development of a prototype system, including the creation of an accompanying mobile application. WS4‐ usability testing through workshop to evaluate functionality and ease of use.

**Result:**

The study is expected to provide both valuable qualitative and quantitative insights into the feasibility and potential of hearables for dementia care, bridging the gap between technological innovation and real‐world application. The study has commenced in January 2025 and is funded by the Imperial College London Dame Julia Higgins Postdoc Collaborative Research Fund

**Conclusion:**

Findings will inform future development, generate potential recommendations, and pilot‐testing, to enhance dementia care pathways and improve outcomes for individuals and their caregivers.

